# The impact of long haul travel on the sleep of elite athletes

**DOI:** 10.1016/j.nbscr.2023.100102

**Published:** 2023-09-20

**Authors:** R. Doherty, S.M. Madigan, A. Nevill, G. Warrington, J.G. Ellis

**Affiliations:** a Sports Lab North West, Atlantic Technological University, Letterkenny Campus, F92 FC93 Donegal, Ireland; b Sport Ireland Institute, National Sports Campus, Abbotstown, D15 PNON, Ireland; c Faculty of Education, Health and Wellbeing, University of Wolverhampton, Walsall Campus, Walsall, WV1 1LY, UK; d Sport and Human Performance Research Centre, Schuman Building, University of Limerick, V94 T9PX, Ireland; e Department of Physical Education and Sport Sciences, University of Limerick, V94 T9PX, Ireland; f Northumbria Centre for Sleep Research, Northumbria University, Newcastle, NE7 7XA, UK

**Keywords:** Sleep, Jet lag, Elite athletes, Eastward travel

## Abstract

In order to manage and implement strategies to alleviate the symptoms of jet lag it is essential to assess the impact of jet lag in athletes. The aim of this study was to assess the impact of long haul eastward travel on elite athletes' (n = 7 elite national track cyclists; male n = 3, and female n = 4) sleep. The athletes’ sleep was monitored before, during and after travel using both actigraphy and self-report measures. Participants wore an activity monitor for 5 days prior to travel, during the long haul travel and 5 days upon arrival at their destination and completed a daily online sleep diary Actigraphy highlighted significant reductions in time in bed, total sleep time and sleep efficiency (%) due to long haul eastward travel, particularly in the 48 h after travel. Sleep diary data exhibited significant reductions in time in bed, total sleep time, sleep efficiency, sleep quality and a significant increase in fatigue going to bed as a result of long haul eastward travel. In order to facilitate the development of interventions to reduce the symptoms and severity of jet lag objective and subjective assessments of sleep should be coupled with assessments of chronotype and perceived sleep need.

## Highlights

• Athletes demanding schedules can require long haul travel across timezones.
• Jet lag is caused by travel across multiple timezones (>3).
• Actigraphy data showed that time in bed, total sleep time and sleep efficiency are reduced for 48 h after travel.
• Sleep diaries showed reductions in time in bed, total sleep time, sleep efficiency and sleep quality and increased fatigue going to bed.
• Long haul travel should be scheduled to allow athletes adequate time to recover from jetlag before competition.

## 1 Introduction

For athletes and support staff the 2021 Tokyo Olympics, involved a considerable eastward journey crossing 7 time zones and up to 24 h travel time, depending on stopovers (Roach and Sargent, 2019). It is vital that the impact of such journeys on sleep is investigated in order to develop strategies to cope with and minimise the effects of travel fatigue and jet lag.

The main determinant of the human ‘body clock’ is situated in the hypothalamus and this master clock becomes synchronised with the external environment, predominately through light/dark cycles (≈24 h ‘circadian rhythm’) (Hastings et al., 2018). The circadian system responds to external and internal signals via the suprachiasmatic nucleus (SCN) which receives environmental cues such as the light-dark cycle and additional information from other areas of the brain in alignment with certain behaviours (e.g. the timing of nutritional intake and/or exercise) (Tahara and Shibata, 2014). Sunlight is the most powerful zeitgeber (time cue) but eating, physical activity, social contact and other lifestyle factors also influence our circadian rhythms (Choy and Salibu, 2011; Reppert and Weaver, 2002; Mistlberger and Skene, 2004).

Humans' sleep schedules are predominantly regulated by exposure to light and the secretion of melatonin which have opposing effects on circadian rhythms [ (Coste and Lagarde, 2009; Sack, 2009)]. Melatonin is secreted by the pineal gland for 10–12 h in the evening and night eliciting a sleep inducing effect (Sack, 2009; Beaumont et al., 2004; Atkinson et al., 2003). The normal synchronicity between the body clock and the environment is disrupted by rapid transmeridian travel with associated negative symptoms that are referred to as ‘jet lag” i.e. a circadian rhythm disorder (Waterhouse et al., 2007). The conditions experienced during long haul travel, such as uncomfortable and confined seating positions, noise levels and stopovers can cause fatigue and disrupt sleep (Fowler et al., 2020). Numerous symptoms commonly manifest as a result of jet lag that impact athletic performance such as fatigue, disturbed sleep, insomnia, excessive daytime sleepiness, decreased alertness, headaches, mood disturbance, decreased motivation, appetite change and gastrointestinal distress (Choy and Salibu, 2011; Diekman and Bose, 2018; Forbes-Robertson et al., 2012; Kolla and Auger, 2011). The consequences of long haul travel on athletes’ sleep and well-being could compromise both training adaptations and/or performance. Keeping athletes healthy in the period prior to important competitions is paramount for optimal performance (Keaney et al., 2019). It has been demonstrated that elite athletes who have fewer injuries and illness and complete ≥80% of planned trainings in the 6 months prior to major events have a greater chance of achieving their predefined goals (Raysmith and Drew, 2016).

### 1.1 Travel fatigue and jet lag

Elite athletes are frequently required to travel both short journeys within time zones (e.g. National championships/leagues, squad camps, etc.) and long haul across time zones (e.g. Olympic Games, World Championships, Continental events, World Student Games, etc.) for training or competitions which causes travel fatigue and jet lag (Biggins et al., 2020; Van Rensburg et al., 2019; Samuels, 2012; Arendt, 2009). The circadian system has to either advance (eastward travel) or delay (westward travel) however, eastward travel is more difficult as endogenous circadian rhythmns have an ∼24.25 h period making it more difficult to delay the circadian system (Van Rensburg et al., 2019). During any long journey travel fatigue develops over time, irrespective of the mode of travel (Samuels, 2012). Travel fatigue is associated with frequent travel while jet lag is associated with time zone displacement (Walsh et al., 2021). Travel fatigue is caused by the demands of travel (e.g. getting to the airport, poor prior sleep, delays and/or flight changes) (Waterhouse et al., 2007). While, jet lag is recognised as a sleep disorder (Thorpy, 2017; Sateia, 2014) that individuals experience after transmeridian travel (across multiple timezones). Travellers with rigid sleep habits appear to be more susceptible to jet lag (Flower et al., 2003). Jet lag involves the sychronisation of the body clock to the new environment (destination time) and it has been suggested that recovery takes place at a rate of approximately 0.5 (west) to 1 day (east) per time zone crossed (Van Rensburg et al., 2019; Sack, 2010). Jet lag is caused by travel across multiple timezones (>3) with resultant desychronisation of the circadian system to the light/dark cycle of the destination (Van Rensburg et al., 2019). Following long-haul east-west travel involving crossing multiple timezones, the circadian system cannot immediately entrain to the new time zone (Wever et al., 1983) and initially upon arrival the circadian system is sychronised with the time zone of departure (Winget et al., 1984). It has been suggested that north-south long haul travel can also cause jet lag due to changes in day length even when no timezones are crossed (Diekman and Bose, 2018). While the human circadian system takes time to adjust to the destination time, jet lag symptoms such as sleep disturbance, daytime sleepiness, difficulty sleeping at night, irritability, gastrointestinal disruption and reduced cognitive and physical performance persist [ (Samuels, 2012), (Edwards et al., 2013)]. As the body clock adjusts to the new time zone, nocturnal sleep improves and the symptoms of daytime fatigue, reduced motivation, poor mental performance begin to dissipate (Edwards et al., 2013). In order to manage and implement strategies to alleviate the symptoms of jet lag it is essential to assess the impact of jet lag in athletes.

## 2 Jet lag and athletes

While the research regarding jet lag and athletes is relatively limited some risk factors for jet lag in athletes have been identified.

### 2.1 Chronotype

Chronotype typically influences individual's reaction to jet lag, morning types typically have less difficultly travelling eastward while evening types usually have less difficulty travelling westward (Baehr et al., 2000; Kerkhof and van Dongen, 1996). It has been suggested that the difference is because morning types cope better with the earlier bedtime and waking time required for eastward travel, while evening types are better able to cope with the delayed bedtime and waking time required for westward travel (Baehr et al., 2000; Kerkhof and van Dongen, 1996; Reilly et al., 2009).

### 2.2 Gender

Males go to sleep earlier and experience less fatigue after crossing multiple time zones during eastward travel (Waterhouse et al., 2002a). The onset of melatonin secretion is earlier for females hence sleep may occur at a later biological time during travel which could impact the effects of jet lag (Cain et al., 2010; Sack et al., 2007).

### 2.3 Travel direction

While it has been suggested that jet lag affects people in a similar way regardless of the direction of travel (Sack, 2010), it is generally accepted that jet lag is more pronounced on eastbound trips (lengthen the day) than on westbound trips (shorten the day) and it is easier to stay awake than to go to ‘bed’ early (Eichner, 2020). Fowler et al. (2017) demonstrated significantly (p < 0.05) higher jet lag symptoms and a greater effect on sleep and performance due to westward versus eastward. Flying west is associated with less jet lag due to ease of sleep onset following a delay in bedtime, likely due to increased fatigue and time awake (Reilly et al., 2009; Lemmer et al., 2002). Recent mathematical modelling of jet lag has highlighted the asymmetry in the severity of jet lag between eastward and westward travel (Diekman and Bose, 2018). While north-south travel may cause jet lag even when no timezones are crossed due to the change in day length (Diekman and Bose, 2018).

### 2.4 Arrival time and number of times zones crossed

Arrival time may impact the severity of jet lag symptoms (Waterhouse et al., 2002a; Fowler et al., 2017). Travel schedules that minimise the intervals between the last full night's sleep before departure and the first full night's sleep upon arrival reduce jet lag (Fowler et al., 2017).

### 2.5 Fitness

Physical fitness has been shown to promote sleep in female athletes (Silva and Paiva, 2018), and has been associated with mental toughness which may improve athletes’ ability to cope with jet lag (Reilly et al., 2009). It has been suggested that the diurnal changes in mental and physical performance can influence other circadian rhythms and could aid the resynchronisation to new timezones (Malhotra, 2017), therefore shifting training schedules prior to departure may reduce the impact of jet lag on performance.

### 2.6 Athletic performance

It has been proposed that there are 3 areas of evidence regarding how jet lag impacts athletic performance (1) the circadian rhythm influences athletic performance (Reilly and Waterhouse, 2009), (2) the negative effects of jet lag (i.e. nocturnal sleep loss, daytime fatigue: reduced motivation and cognitive performance) which can affect athletic performance (Walsh et al., 2021): and (3) the results of games/matches (i.e. win-loss records) (Huyghe et al., 2022; Roy and Forest, 2018; Jehue et al., 1993).

### 2.7 Cognitive performance

Jet lag, the resultant sleep loss and/or disruptions to the circadian rhythmn can alter cognitive function (Lee and Galvez, 2012). Mood and complex cognitive tasks deteriorate more quickly than simpler tasks (Reilly et al., 2008). Mental performance is adversely impacted by sleep loss which is directly related to jet lag and crossing multiple timezones (Reilly et al., 1997; Waterhouse et al., 2002b).

Further research is necessary to assess the impact of jet lag on athletes’ sleep as it has the potential to negatively affect both health and performance (Van Rensburg et al., 2019; Lee and Galvez, 2012; Reilly et al., 2008; Waterhouse et al., 2002b). The aim of this study was to assess the impact of long-haul travel and jet lag on the sleep of elite athletes. In order to assess the impact of long-haul travel and jet lag on their sleep the athletes were monitored before, during and after travel. Therefore, the objectives of the study were to: 1) assess baseline sleep, 2) the impact of travel on their subjective and objective sleep measures and 3) assess sleep upon arrival at the destination.

## 3 Methodology

### 3.1 Participants

An entire elite international track cycling squad (n = 7; n = 3 males and n = 4 females) were recruited. Written informed consent was provided prior to data collection. The athletes were regarded as elite in line with published definitions i.e. members of a national/professional team (Swann et al., 2015).

### 3.2 Measures

**Fig. 1:**
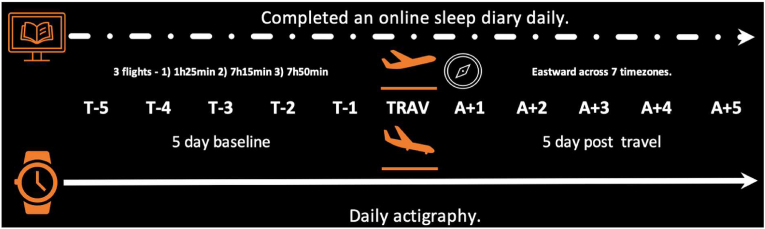
Schematic of study design.

All procedures were approved by the research ethics committee of the Faculty of Health and Life Sciences, Northumbria University. Participants wore an activity monitor (Philips Respironics ActiWatch 2, Phillips, Amsterdam, Netherlands), for 5 days prior to travel, during the long haul travel and 5 days upon arrival at their destination and completed a daily online sleep diary (see Fig. 1). The athletes did not receive any sleep education, were not employing any sleep/jet lag countermeasures and travelled in economy class for all flights which involved travel across 7 time zones.

Flight 1: Majorca to Madrid (1 h 25 min)

Transit: 2 h.

Flight 2: Madrid to Dubai (7 h 15 min)

Transit: 3 h.

Flight 3: Dubai to Hong Kong (7 h 50 min) Travel time: 21.5 h.

### 3.3 Actigraphy

Actigraphy is a valid and reliable alternative to PSG for sleep assessment which is widely used in athletic populations (Halson, 2019). In contrast to polysomnography, actigraphy offers a cost effective and field-based assessment of sleep which can be implemented in ‘real-world’ settings with athletes (Taylor et al., 2017). The following sleep indices were derived from the actigraphy data (see Table 2).

• Time in bed (min): the time spent in bed between going to bed and getting up
• Total sleep time (min): the amount of time spent asleep
• Sleep onset latency (min): the amount of time it took to get to sleep
• Sleep efficiency (%): sleep duration expressed as a percentage of time in bed
• Wake after sleep onset (min): time spent awake between start and end of sleep
• Awakenings (#): number of awakenings.

### 3.4 Consensus sleep dairy – core

Participants were instructed to complete an online version of the Consensus Sleep Diary – Core (CSD-C) a standardised research sleep diary for each night's sleep for the duration of the study (Carney et al., 2012). The CSD-C included 8 items e.g. bed time, time it took to fall asleep, number of awakenings, duration of awakenings, time of final awakening, time the respondent got out of bed, and a Likert scale self-report rating of sleep quality (Maich et al., 2016). Participants were also instructed to record levels of fatigue both going to bed and in the morning on a likert scale (1 = Completely exhausted to 7 = Fully Alert) (see Table 2).

### 3.5 Data analysis

All data was analysed using the Statistical Package for the Social Sciences (SPSS Version 26, IBM Corporation) and Jamovi Version 1.6.18 (The Jamovi Project). Q-Q plots and Shapiro-Wilks tests were used to assess the distribution of data. Frequency distribution and descriptive statistics were used to present findings with all data being presented as mean ± standard deviation, mode and/or frequency (Thomas et al., 2015). One way ANOVA, Kruskal-Wallis tests, post hoc analysis and Linear Regression were used to assess the impact of jet lag (day) on the athletes’ sleep. To examine the difference in objective sleep measures due to long haul travel, linear mixed models were fitted, considering each sleep variable and day, their interaction as fixed effects, with participant as a random effect.

## 4 Results

At baseline participants’ age and body mass were recorded, they were given an Actiwatch and began daily sleep diary completion. A Chi square analysis demonstrated no significant differences between the participants for gender (X^2^ [1, n = 77] = 1.57, p = 0.21).

Participants’ sleep was assessed before, during and after long haul travel using the ActiWatch 2 wrist worn activity monitor (see Table 1).

**Table 1:** Summary of actigraphy assessments (mean ± SD).

Day	TIB (min)	TST (min)	SOL (min)	SE (%)	WASO (min)	Awakenings (#)	Key
**T-5**	650 ± 138	567 ± 126	21.2 ± 38.8	87.4 ± 9.26	37.5 ± 36.2	33.9 ± 25.1	T-5: 5 days before travel
**T-4**	523 ± 146	426 ± 138	44.4 ± 48.3	80.3 ± 11.8	34.9 ± 13.6	36.9 ± 14.1	T-4: 4 days before travel
**T-3**	564 ± 74.9	442 ± 114	51.1 ± 65.9	77.3 ± 10.8	48 ± 34.4	44.3 ± 18.7	T-3: 3 days before travel
**T-2**	577 ± 116	489 ± 125	35.4 ± 40.6	82.7 ± 5.29	39.5 ± 20.3	38.6 ± 17.6	T-2: 2 days before travel
**T-1**	570 ± 90	472 ± 43.7	28.2 ± 47.8	83.9 ± 7.83	34.3 ± 16.2	40.4 ± 13.5	T-1: day before travel
**Trav**	363 ± 124	211 ± 77	64.4 ± 42.8	59.8 ± 15.9	27 ± 12.6	20.6 ± 11.1	Trav: travel day
**A+1**	384 ± 129	237 ± 83.7	62.7 ± 63.9	65.5 ± 19.9	46.9 ± 41.6	28 ± 23.3	A+1: day after arrival
**A+2**	574 ± 191	463 ± 154	3.43 ± 7.28	84.1 ± 8.44	32.9 ± 19.9	32.7 ± 8.94	A+2: 2 days after arrival
**A+3**	487 ± 85.2	410 ± 91.5	23.4 ± 13.4	85.3 ± 5.91	30.9 ± 18.3	30.4 ± 14	A+3: 3 days after arrival
**A+4**	567 ± 33.8	500 ± 33.7	20.2 ± 13.2	88.7 ± 3.96	35.1 ± 13.4	40 ± 12.9	A+4: 4 days after arrival
**A+5**	595 ± 42	434 ± 176	51.2 ± 54.3	84.4 ± 8.42	37.4 ± 18.5	43.3 ± 20.7	A+5: 5 days after arrival

### 4.1 Actigraphy

#### 4.1.1 Time in bed (min)

**Fig. 2:**
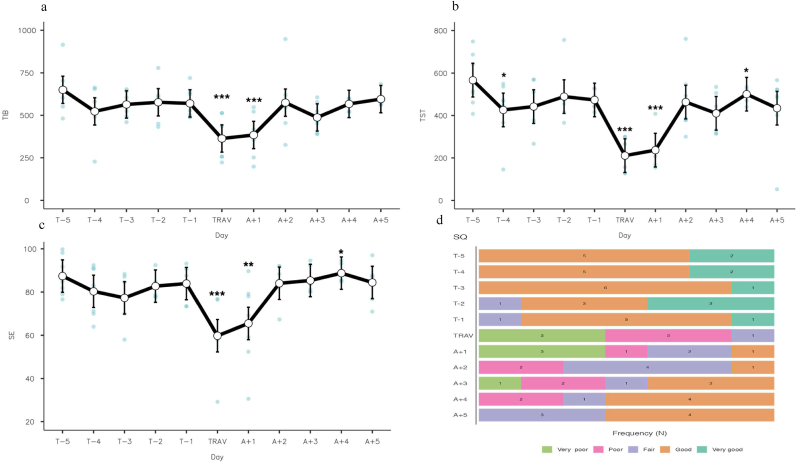
Changes in Actigraphy total sleep time, time in bed, sleep efficiency (%) and self-reported sleep quality by day.

There were statistically significant differences for TIB based on day. A mixed model was used to assess the impact of day on TIB, a significant regression was found (F (10, 77) = 4.78; p < 0.001; R^2^ = 0.386). TIB was significantly reduced by 213 min (p < 0.001, SE 44.2, 95% CI [−299.7 to −126.5]) on TRAV and 157 min (p < 0.001, SE 43.6, 95% CI [−242.4 to −71.6]) on A+1 compared to all other days (see Fig. 2a).

#### 4.1.2 Total sleep time (min)

TST significantly varied based on day. A mixed model was used to assess the impact of day on TST, a significant regression was found (F (10, 70) = 7.36; p < 0.001; R^2^ = 0.495). TST was significantly reduced by 140 min (p = 0.015, SE 56, 95% CI [−250.23 to −30.6]), on T-4, 268 min (p < 0.001, SE 43.6, 95% CI [−353.68.4 to −183.6]) on TRAV, 197 min (p < 0.001, SE 42.8, 95% CI [−281.79 to −114.1]) on A+1 and increased by 87 min (p = 0.04, SE 41.8, 95% CI 5.4 to −169.2]) on A+4 compared to all other days (see Fig. 2b).

#### 4.1.3 Sleep latency

While SOL increased on TRAV and A+1, a one way ANOVA (Kruskal-Wallis) demonstrated there were no statistically significant differences between SOL scores.

#### 4.1.4 Sleep efficiency

SE significantly varied based on day, a mixed model was used to assess the impact of day on SE, a significant regression was found (F (10, 70) = 6.11; p < 0.001; R^2^ = 0.452). SE was significantly reduced by 22.63% (p < 0.001, SE 4.08, 95% CI [−30.5 to −14.56]) on TRAV, 13.12% (p = 0.002, SE 4.02, 95% CI [−20.99 to −5.24]) on A+1 and increased by 10.27% (p = 0.011, SE 3.92, 95% CI 2.58 to −17.96]) on A+4 compared to all other days (see Fig. 2c).

#### 4.1.5 Awakenings

There was no statistically significant difference between WASO scores by day.

## 5 Sleep diary

Participants’ self-reported their sleep and fatigue levels both before bed and in the morning after waking before, during and after long haul travel using online sleep diaries (see Table 2).

**Table 2:** Summary of sleep dairy responses (mean ± SD; mode).

Day	TIB (min)	TST (min)	SOL (min)	SE (%)	WASO (min)	Awakenings (#)	Fatigue (Bedtime)	Fatigue (Morning)	SQ
**T-5**	666 ± 139	624 ± 156	12.1 ± 12.9	93 ± 5.67	30.1 ± 26.6	5.14 ± 3.48	3 (Moderate)	3 (Moderate)	4 (Good)
**T-4**	536 ± 140	474 ± 132	26.4 ± 27.3	88.2 ± 6.03	35.1 ± 14.2	4.43 ± 1.81	3 (Moderate)	3 (Moderate)	4 (Good)
**T-3**	560 ± 80.8	500 ± 100	29.3 ± 41.8	88.6 ± 9.12	30.6 ± 23.8	4.29 ± 1.25	3 (Moderate)	3 (Moderate)	4 (Good)
**T-2**	587 ± 126	530 ± 135	21.7 ± 23.6	89.8 ± 5.39	35.4 ± 23.7	4.29 ± 1.7	3 (Moderate)	5 (Somewhat fresh)	4 (Good)
**T-1**	558 ± 70.3	504 ± 54.1	24.6 ± 43.4	90.7 ± 6.39	29.7 ± 13.5	5 ± 2.89	3 (Moderate)	3 (Moderate)	4 (Good)
**Trav**	426 ± 119	357 ± 116	44.3 ± 33	82.9 ± 9.31	24.1 ± 12.5	4.29 ± 2.63	1 (Exhausted)	1 (Exhausted)	1 (Very Poor)
**A+1**	405 ± 140	327 ± 123	42.1 ± 28.8	81 ± 11.2	36.6 ± 38.3	3.29 ± 2.69	2 (Very tired)	2 (Very tired)	1 (Very poor)
**A+2**	591 ± 183	558 ± 163	5.71 ± 7.32	94.7 ± 2.03	28.1 ± 21.1	5.43 ± 3.26	2 (Very tired)	2 (Very tired)	3 (Fair)
**A+3**	508 ± 75.8	460 ± 82.6	19.3 ± 12.4	90.4 ± 5.16	28.4 ± 16.3	4.57 ± 2.15	3 (Moderate)	3 (Moderate)	4 (Good)
**A+4**	578 ± 28.8	528 ± 38	20.7 ± 15.9	91.4 ± 3.09	28.9 ± 21.7	4.14 ± 3.13	3 (Moderate)	4 (A little tired)	4 (Good)
**A+5**	615 ± 48.4	542 ± 50.8	47.1 ± 48.3	88.3 ± 7.87	26.3 ± 14	3.57 ± 2.07	4 (A little tired)	3 (Moderate)	4 (Good)

### 5.1 Sleep dairy

There were no statistically significant differences for SOL and awakenings. Sleep diary TIB varied significantly by day (X^2^ [1, n = 10] = 25.21, p ≤ 0.005; ε^2^ = 0.33). Dwass-Steel-Critchlow-Fligner post hoc analysis indicated no pairwise comparisons. A linear regression model was used to assess the impact of day on sleep diary TIB, there was a significant effect found for TIB × day (F (10, 66) = 3.24; p ≤ 0.002; R^2^ = 0.228).

Sleep diary TST significantly varied based on day (X^2^ [1, n = 10] = 28.08, p ≤ 0.002; ε^2^ = 0.37). Dwass-Steel-Critchlow-Fligner post hoc analysis indicated no pairwise comparisons. A linear regression model was used to assess the impact of day on sleep diary TST, there was a significant effect found for TST × day (F (10, 66) = 4.1; p ≤ 0.01; R^2^ = 0.38).

Sleep dairy SE % significantly varied based on day, a one way ANOVA (Kruskal-Wallis) (X^2^ [1, n = 10] = 19.1, p ≤ 0.04; ε^2^ = 0.25). Dwass-Steel-Critchlow-Fligner post hoc analysis indicated no pairwise comparisons. A linear regression model was used to assess the impact of day on fatigue going to bed. There was a significant effect found for SE % x day (F (10, 66) = 2.32; p = 0.021; R^2^ = 0.26).

### 5.2 Fatigue

There was a statistically significant difference between fatigue going to bed scores by day (X^2^ [1, n = 10] = 40.1, p ≤ 0.001; ε^2^ = 0.528). Dwass-Steel-Critchlow-Fligner post hoc analysis indicated significant differences between TRAV and T-5, T-4, T-3, T-2, T-1, A+4 and A+5. A linear regression model was used to assess the impact of day on fatigue going to bed. There was a significant effect found for fatigue going to bed x day (F (10, 66) = 8.22; p ≤ 0.001; R^2^ = 0.555). There was no statistically significant difference between fatigue in the morning scores by day (X^2^ [1, n = 10] = 10.8, p = 376; ε^2^ = 0.142).

### 5.3 Sleep quality

SQ significantly varied based on day, there was a statistically significant difference between SQ by day (X^2^ [1, n = 10] = 3.15, p; ε^2^ = 0.588). Dwass-Steel-Critchlow-Fligner post hoc analysis indicated significant differences between TRAV and T-5, T-4 and T-3 (see Fig. 2d). A linear regression model was used to assess the impact of day on sleep quality, there was a significant effect (F (10, 66) = 9.54; p ≤ 0.001; R^2^ = 0.529).

## 6 Discussion

The aim of the current study was to assess the sleep of elite athletes before long haul travel and the subsequent impact on sleep whilst in transit and upon arrival at their destination, in order to provide a) an indicator of what sleep parameters are affected by competition travel which will inform what sleep parameters an intervention is needed to address and b) an overview of when the intervention may be most beneficial over the course of competition travel. Unless the negative effects of both long haul travel and the resultant jet lag upon arrival can be managed or minimised then athlete health, well-being and performance are likely to be affected (Fowler et al., 2020). A recent study investigating the impact of jet lag on the sleep of an elite Rugby union team (n = 37) following long haul westward travel, showed TST reduced during and after travel but returned to normal after 2 days of adjustment upon arrival (Smithies et al., 2021). A strength of this study is the fact that the athletes had not received any sleep education and did not employ any sleep strategies or jet lag counter measures during travel, and the findings could be used to inform future practice. The results of the current study indicated that the following sleep parameters: actigraphy derived TIB, TST and SE and sleep diary derived TIB, TST, fatigue going to bed and SQ were significantly negatively impacted by long haul travel. No significant effects were observed for long haul travel on WASO, number of awakenings and fatigue in the morning. In the current study, the majority of sleep disruption was observed during the initial 48 h post travel period, this is consistent with previous research that highlighted that the greatest disruption occurred in the first 48 h period following travel (Smithies et al., 2021; Stevens et al., 2018). It is clear from the current study and previous research that teams/athletes should complete long-haul travel 5–6 days prior to competition to allow sufficient time for athletes to recover from travel fatigue and jet lag.

Actigraphy measured TIB was significantly reduced as a result of travel (363 ± 124 min) and on the first night (384 ± 129) after arrival, these findings were also demonstrated in the sleep dairy TIB for TRAV and A+1. Indeed, mean TIB for TRAV and A+1 were less than current sleep recommendations (7–9 h) for adults (Hirshkowitz et al., 2015), hence there was not enough TIB to gain adequate total sleep times. The negative impact of long haul travel on TIB on TRAV and A+1 was observed for all athletes. It has been suggested that sleep can be difficult on long haul flights due to an uncomfortable upright seating/sleeping position, light, noise and the environment and routine on the flight (Fowler et al., 2020; Stevens et al., 2018; Roach et al., 2018). It is common practice to advise athletes to schedule their sleep on a long haul flight to coincide with night time at their destination to prevent their sleep/wake cycle from ‘anchoring’ to the departure timezone (Roach and Sargent, 2019; Reilly et al., 2008). However, it has recently been recommended that in order to maximise sleep and minimise the impact of jetlag athletes should sleep during a ‘sleep window’, that corresponds to night-time at the place of departure (i.e. during biological night time when the circadian system is promoting sleep) (12). Fowler et al. (2020) demonstrated that this approach reduced sleep disruption during travel but also had no negative effect on sleep or jet lag symptoms post travel.

A range of 7–9 h sleep has been suggested as appropriate for healthy adults (Hirshkowitz et al., 2015; Watson et al., 2015), however, athletes may require more sleep due to the physical and psychological demands of their sport associated with training and competition (Van Rensburg et al., 2019; Walsh et al., 2021). Actigraphy TST was significantly reduced on TRAV (211 ± 77 min) and A+1 (237 ± 83.7 min) compared to the other days. Sleep diary TST was also reduced on TRAV and A+1. While TST during baseline was adequate, TST was less than current sleep recommendations (i.e. 7-9 h) [ (Hirshkowitz et al., 2015), (Watson et al., 2015)], on TRAV, A+1 and A+3, therefore it was not possible for the athletes to get adequate sleep. Previous research has suggested that sleep duration was decreased during travel but increased on the first night after arrival (Stevens et al., 2018). This contrasts with the current study whereby TST was significantly reduced on TRAV and A+1. Indeed, for all athletes the range of sleep time on both TRAV (127–300 min) and A+1 (155–408 min) was below the recommended range for athletes (7–9 h) (Hirshkowitz et al., 2015), potentially chronotype had a role to play i.e. being unable to delay wake time. Lastella et al. (2014) noted reduced TST (6.6 ± 1.3 h per night compared to baseline 7.5 ± 1.3 h) immediately following long haul eastward travel crossing 8 timezones (Sydney to Denver, Colorado) however, it was noted that the altitude of the destination may have been a confounding factor.

Sleep preservation is an important strategy in the management of jet lag (Arendt, 2009). Sleep hygiene has been shown to help healthy adults overcome travel fatigue following 24-h of simulated international travel and while good sleep hygiene may not phase shift the circadian system, some sleep habits (e.g. use of electronic devices) can induce phase shifts (Fowler et al., 2015). Long haul and overnight travel are most likely to cause disrupted sleep patterns and adversely affect both sleep quality and quantity (Fowler et al., 2016). Similar to the current study, an investigation of the impact of long haul international travel (18 h, -4 h timezone shift) in elite male football players (n = 15) on sleep highlighted significantly (p > 0.05) reduced sleep duration and sleep efficiency during and immediately following travel (Fullagar et al., 2016).

In athletic populations when sleep is reduced to <7 h cognitive performance (i.e. alertness, reaction time, memory and decision making) and physical performance and injury risk are adversely affected (Charest and Grandner, 2020; Laux et al., 2015; Le Meur et al., 2013). Sleep loss is central to the negative impact of jet lag on performance, daytime fatigue and gastrointestinal comfort (Van Rensburg et al., 2019; Fowler et al., 2017). A recent study in Rugby 7's players (n = 17; aged 25.4 ± 5.1 year) during the competitive season indicated that long haul travel had a negligible impact on actigraphy assessed sleep quantity and quality in the immediate period after travel, however it was suggested that the team had implemented efficient and robust travel strategies (Leduc et al., 2021), it must also be noted that given their age this group may have more plasticity in their sleep system (Ohayon et al., 2017). Previous research in Rugby 7's has indicated that a simple dose response relationship does not exist between travel duration, competition demands, and number of time zones crossed and that highly individual responses are observed reinforcing the need for individualised support (Fowler et al., 2019). The role of sleep is increasingly recognised in terms of both general health and athletic performance (Jukic et al., 2021) and it is clear that in the current study jetlag had a significant negative impact for 48 h post travel. Athlete sleep need could be assessed subjectively, indeed a recent study in elite athletes (n = 175) included a self-report assessment of sleep need, athletes reported an average sleep need of 8.3 ± 0.9 h (Sargent et al., 2021). An individualised approach incorporating an assessment of the athlete's perceived sleep need should be employed. As such, a one size fits all approach to athlete sleep recommendations, sleep hygiene strategies and interventions to reduce the symptoms and impact of jet lag may be inadequate (Arendt, 2009).

A common symptom of jetlag following long haul eastward travel is sleep disruption due to delayed SOL (Fowler et al., 2017; Reilly et al., 2009). In the current study SOL significantly increased on TRAV (64.4 ± 42.8min) and A+1 (62.7 ± 63.9min), however, SOL exceeded 20 min on all days except A+2 (3.43 ± 7.48 min), which may account for the lack of significant pairwise comparisons in SOL by day. Long SOL has previously been reported as an issue in elite athletes related to sleep inadequacy in general, not just during travel, which can be attributed to insomnia (Tuomilehto et al., 2016; Schaal et al., 2011).

Reduced TIB and TST due to travel and jet lag resulted in poorer SE% <85% (i.e. poor sleep), it was observed on T-4 (80.3%), T-3 (77.3%), T-2 (82.7%), T-1 (83.9%), TRAV (59.8%), A+1 (65.5%), A+2 (84.1%) and A+5 (84.4%). However, SE was reduced in the 48 h period post travel then began to return to baseline levels, this was consistent with previous research that demonstrated significant reductions in SE both during travel and the subsequent 48 h (Fullagar et al., 2016). It has recently been shown that SE is reduced following long haul travel for approximately 72 h (Fowler et al., 2020).

In the current study there was a significant difference between fatigue going to bed between TRAV and T-5, T-4, T-3, T-2, T-1, A+4 and A+5. There was no significant impact observed for jet lag on awakenings, number of awakenings or fatigue in the morning. It has previously been suggested that athletes report more fatigue during long haul travel than following training and competition (Calleja-Gonzalez et al., 2020). In order to support athlete health, travel experience, and performance the implementation of sleep hygiene practices, nutritional programming, load monitoring, fatigue reporting and recovery practices are paramount particularly during travel (Calleja-Gonzalez et al., 2020).

There was a significant negative effect on SQ as a result of long haul travel with TRAV being significantly different than T-5, T-4 and T-3. Travel has previously been indicated as a sport related risk factor for inadequate and/or non-restorative sleep (Nédélec et al., 2018; Gupta et al., 2017). Recovery from jet lag and subsequent improvement in sleep quality requires resynchronisation of the circadian rhythm to the destination light-dark cycle (Roach and Sargent, 2019). Following eastward travel, the circadian rhythm requires time to advance which is generally more difficult, it has previously been suggested that resynchronisation following eastward travel requires 1 day per timezone crossed (Van Rensburg et al., 2019). However, in the current study the negative effects of jet lag began to dissipate after 48 h.

### 6.1 Limitations

The fact that chronotype was not assessed in the current study is a limitation as jetlag following eastward travel may be less pronounced in morning types (32; 30; 31). As discussed, previous research has suggested a skew towards morningness in elite athletes (Lastella et al., 2014; Bender et al., 2019; Silva et al., 2012) which might partially explain the findings of the current study. It must also be noted that future studies should include measures of performance to assess the impact of jet lag on athlete performance. However, the aim of the study was not to assess mediators or moderators of jetlag but to identify the sleep parameters that are most affected by competition travel.

A further limitation of the current study is the small sample size (n = 7) however, it must be noted that an entire national squad completed the study. The rareness of elite athletes makes recruiting such participants in large numbers difficult (Sands et al., 2019).

### 6.2 Practical applications

These findings support the suggestion that when athletes are required to travel across multiple timezones, it is essential to allow enough time between arrival at the destination and the start of the event for athletes to recover and adjust to the new time zone (Van Rensburg et al., 2019; Lastella et al., 2014). In the current study TST, TIB, SE (%), fatigue going to bed and SQ were significantly adversely affected both during and after (+48 h) long haul travel, all measures began to return to baseline values by A+3. These findings are similar to a recent study of Rugby players which suggested that long haul travel should completed at least 6 days prior to competition (Smithies et al., 2021). The development and employment of individualised jet lag interventions, including sleep hygiene practices, may help reduce jet lag symptoms and severity (Van Rensburg et al., 2019; Fowler et al., 2015, 2016).

## 7 Conclusion

Actigraphy highlighted significant reductions in time in bed, total sleep time and sleep efficiency (%) due to long haul eastward travel, particularly in the 48 h after travel, after this period all measures began to return towards baseline. Sleep diary data exhibited significant reductions in time in bed, total sleep time, sleep efficiency, sleep quality and a significant increase in fatigue going to bed as a result of long haul eastward travel. The level of sleep disruption observed in the 48 h post eastward travel in the current study could negatively impact both athlete health and performance. In order to facilitate the development of interventions to reduce the symptoms and severity of jet lag objective and subjective assessments of sleep should be coupled with assessments of chronotype and perceived sleep need.

## Credit author statement

Rónán Doherty: Conceptualisation, Methodology, Resources, Data Curation, Formal Analysis, Investigation, Writing – original draft, Writing – Review & Editing, Visualisation. Sharon Madigan: Supervision, Resources, Writing – Review & Editing. Alan Nevill: Formal Analysis. Giles Warrington: Supervision, Writing – Review & Editing Jason G Ellis: Supervision, Conceptualisation, Methodology, Writing – Review & Editing.

## Declaration of competing interest

The authors declare that they have no known competing financial interests or personal relationships that could have appeared to influence the work reported in this paper.

## Data Availability

Data will be made available on request.
